# First Crystal Structure of Bacterial Oligopeptidase B in an Intermediate State: The Roles of the Hinge Region Modification and Spermine

**DOI:** 10.3390/biology10101021

**Published:** 2021-10-09

**Authors:** Dmitry E. Petrenko, Vladimir I. Timofeev, Vladimir V. Britikov, Elena V. Britikova, Sergey Y. Kleymenov, Anna V. Vlaskina, Inna P. Kuranova, Anna G. Mikhailova, Tatiana V. Rakitina

**Affiliations:** 1 National Research Center “Kurchatov Institute”, 123182 Moscow, Russia; dmitry.e.petrenko@gmail.com (D.E.P.); annavlaskina@yandex.ru (A.V.V.); 2 Shemyakin-Ovchinnikov Institute of Bioorganic Chemistry, RAS, 117997 Moscow, Russia; anna.g.mikhailova@gmail.com; 3 Federal Scientific Research Center “Crystallography and Photonics”, RAS, 119333 Moscow, Russia; inna@crys.ras.ru; 4 Institute of Bioorganic Chemistry, National Academy of Sciences of Belarus, 220141 Minsk, Belarus; britikov@iboch.by (V.V.B.); britikova@iboch.by (E.V.B.); 5 Bach Institute of Biochemistry, Federal Research Center “Fundamentals of Biotechnology”, RAS, 119071 Moscow, Russia; s.yu.kleymenov@gmail.com; 6 Koltzov Institute of Developmental Biology, RAS, 119334 Moscow, Russia

**Keywords:** prolyloligopeptidase, oligopeptidase B, *Serratia proteomaculans*, crystal structure, intermediate state, hinge region, spermine, small-angle X-ray scattering

## Abstract

**Simple Summary:**

Oligopeptidase B is a two-domain, trypsin-like peptidase from parasitic protozoa and bacteria which belongs to the least studied group of prolyloligopeptidases. In this study, we describe for the first time a crystal structure of bacterial oligopeptidase B and compare it with those of protozoan oligopeptidases B and related prolyloligopeptidases. The enzyme was crystallized in the presence of spermine and contained a modified sequence of the interdomain linker. Both factors were key for crystallization. The structure showed an uncommon intermediate conformation with a domain arrangement intermediate between open and closed conformations found in the crystals of ligand-free and inhibitor-bound prolyloligopeptidases, respectively. To evaluate the impact of the modification and spermine in the obtained conformation, small-angle X-ray scattering was applied, which showed that in solution wild-type enzymes adopt the open conformation and spermine causes a transition to the intermediate state, while the modification is associated with a partial transition. We suggest that spermine-dependent conformational transition replicates the behavior of the enzyme in bacterial cells and the intermediate state, which is rarely detected in vitro, and might be widely distributed in vivo, and so should be considered during computational studies, including those aimed wanting to develop the small molecule inhibitors targeting prolyloligopeptidases.

**Abstract:**

Oligopeptidase B (OpB) is a two-domain, trypsin-like serine peptidase belonging to the S9 prolyloligopeptidase (POP) family. Two domains are linked by a hinge region that participates in the transition of the enzyme between two major states—closed and open—in which domains and residues of the catalytic triad are located close to each other and separated, respectively. In this study, we described, for the first time, a structure of OpB from bacteria obtained for an enzyme from *Serratia proteomaculans* with a modified hinge region (PSPmod). PSPmod was crystallized in a conformation characterized by a disruption of the catalytic triad together with a domain arrangement intermediate between open and closed states found in crystals of ligand-free and inhibitor-bound POP, respectively. Two additional derivatives of PSPmod were crystallized in the same conformation. Neither wild-type PSP nor its corresponding mutated variants were susceptible to crystallization, indicating that the hinge region modification was key in the crystallization process. The second key factor was suggested to be polyamine spermine since all crystals were grown in its presence. The influences of the hinge region modification and spermine on the conformational state of PSP in solution were evaluated by small-angle X-ray scattering. SAXS showed that, in solution, wild-type PSP adopted the open state, spermine caused the conformational transition to the intermediate state, and spermine-free PSPmod contained molecules in the open and intermediate conformations in dynamic equilibrium.

## 1. Introduction

Oligopeptidase B (OpB, EC 3.4.21.83) is a two-domain, trypsin-like serine peptidase belonging to the S9 family of prolyloligopeptidase (POP), which also includes prolylendopeptidase (PEP, EC 3.4.21.26), alternatively called the namesake of the family (POP), acylaminoacylpeptidase (AAP, EC 3.4.19.1) and dipeptidylpeptidase IV (DPP, EC 3.4.14.5) [1]. The POP family members are distributed into subfamilies S9A–S9C according to their substrate specificities [2]. OpB and PEP (S9A) are endopeptidases that cleave peptide bonds on the carboxyl side of the basic amino acid residues and proline, respectively; DPP (S9B) possess specificity toward proline and cleave dipeptides from the N-terminus of oligopeptides, while AAP (S9C) remove N-acetylated proline from the N-termini.

OpB is the least studied group in the S9 family. In contrast to other POP, OpB was found only in prokaryotes, ancient unicellular eukaryotes and some higher plants [3]. OpB are considered important virulence factors of protozoan infections caused by *Trypanosoma* and *Leishmania* spp. and putative therapeutic targets for the treatment of the corresponding diseases and/or development of vaccines [4,5,6,7]. Although the first described OpB was an enzyme from *Escherichia coli* (EcOpB) [8], currently, the physiological role, structure, and pharmacological value of bacterial OpB are much less studied than those of protozoan OpB. Thus far, no structures have been described. At the same time, a role of OpB in bacterial resistance to certain types of antimicrobial peptides, which are considered a promising alternative to antibiotic therapy, has been proposed [9], which requires increased efforts to expand our knowledge about structure–functional relationships in bacterial OpB.

One of the main structural characteristics of POP is the arrangement between its catalytic α/β hydrolase domain, where the amino acid residues Ser, Asp and His of the catalytic triad are located, and the β-propeller domain, which restricts access to the active site for substrates larger than 3 kDa [10,11]. The domains are linked by a hinge region that allows the transition of the enzyme between an open, closed, and intermediate conformational states. In the closed (active) state, the domains and residues of the catalytic triad are located close to each other, which allows the catalysis to proceed. In the open (inactive) state, the domains and residues of the catalytic triad are separated, which prevents the catalysis but facilitates the entry of the substrate into the active site buried in the interdomain cleft. The intermediate state combines a disrupted catalytic triad of the open state with a domain closeness resembling the closed state.

Open and closed states were detected in crystals of ligand-free and inhibitor-bound bacterial PEP from *Sphingomonas capsulate*, *Myxococcus xanthus*, and *Aeromonas punctate* (ApPEP), respectively [12,13]. In contrast, different monomers of ligand-free dimeric AAP from archaea *Aeropyrum pernix* adopted either conformation independently of one another [14,15]. In the first case, such interdomain dynamics indicates an induced fit mechanism of substrate binding; in the second, a conformational selection is indicated. Only closed states were found in the crystal structures of both ligand-free and substrate/inhibitor-bound forms of mammalian PEP, while the importance of interdomain dynamics was confirmed by engineering of artificial interdomain disulfide bridges [16] and ^15^N relaxation NMR experiments [17]. Various potential substrate access routes to the active center were proposed: one—through the central pore at the top of the β-propeller [18,19], another—through surface loop separation at the interdomain interface [20,21,22]; the interdomain movements identical to those of bacterial PEP were also considered [23]. An intermediate state was detected only twice: in the crystal structures of catalytically impaired macrocyclases from *Galerina marginata* (GmPEP) in complexes with macrocyclization substrates, where it was attributed to the mutations [24], and in structures of archaeal PEP from *Pyrococcus furiosus* (PfPEP) [25].

Three structures of protozoan OpB are currently available. Closed states were observed in two structures of the enzymes from *L. major* (LmOpB) and *T. brucei* (TbOpB) in complexes with peptide inhibitor, transition state analog, antipain (N-carboxyl-FRVRgl) [26,27], and the open state was found in the structure of TbOpB in ligand-free form [26]. This allowed a comparative structural analysis of the open and closed states of protozoan OpB, bacterial PEP and archaeal AAP [26]. A common mechanism of catalytic activation for all three branches of POP was suggested, which highlighted the importance of the interdomain interface and particularly of one of the interdomain salt bridges (SB1 in TbOpB) in the transition of the enzymes between two states [26]. It is intriguing that the residues forming this SB1 were not conserved in γ-proteobacterial OpB [28,29], including the well-studied enzymes from *E.* *coli* [30], *Salmonella enterica* [31] and *Serratia proteomaculans* [32]. This difference strongly suggests there is no direct transfer of the activation mechanism proposed for protozoan OpB to the bacterial enzymes and requires applications of the structural data obtained for OpB from bacteria to elucidate the mechanisms underlying their catalytic activation.

In this study, we described for the first time the structures of bacterial OpB from *S. proteomaculans* (PSP) obtained by X-ray for an enzyme with a modified hinge region (PSPmod) and two of its derivatives. The enzymes were crystallized in the presence of spermine and adopted uncommon intermediate states in the crystal lattices. At the same time, according to small-angle X-ray scattering (SAXS) wild-type PSP adopts an open state in solution; spermine causes its transition to the intermediate state, while PSPmod contained molecules in the open and intermediate states in dynamic equilibrium. The data obtained indicate that the intermediate state, which is rarely found in the crystal structures of enzymes of the POP family, may be much more common in vivo.

## 2. Materials and Methods

### 2.1. Mutagenesis

Easy single-primer site-directed mutagenesis was performed as described in [33]. Oligonucleotide mutagenesis primer (5′-GAG ATG GTG GCG CGC GAG AAC CTG TAT TTC CAA TCG GTG CCT TAT GTC CG-3′) and check-primer (5′-AGA TGG TGG CGC GCG AG-3′), designed for the selection of mutant clones, were synthetized in (Evrogen, Moscow, Russia). Eighteen cycles of polymerase chain reaction (PCR) were performed on the templates of the PSP- and PSP-E125A-expressing plasmids [28] using Tersus Plus PCR kit (Evrogen, Moscow, Russia) according to the manufacturer’s recommendations. The PCR products were treated with DpnI endonuclease (Thermo Fisher Scientific, MA, USA), which digested the parental DNA template, and then transformed into E. coli Match1 competent cells. The mutant clones were selected by PCR performed directly on colonies using Taq DNA polymerase (Evrogen, Moscow, Russia) and check primer with T7 reverse universal primer. Plasmid DNA purified from mutant clones was sequenced to ensure the absence of random mutations associated with PCR. The second run of mutagenesis was performed for preparations of PSPmodE75 on the template of the PSPmod-expressing plasmid. All mutated proteins were verified by Maldi-TOF mass spectrometry.

### 2.2. Recombinant Proteins Purification and Characterization

Proteins were expressed in *E. coli* BL21(DE3) (Novagen, Madison, WI, USA) and purified as described in [32]. Protein sizes and purities were checked by sodium dodecyl sulfate-polyacrylamide gel electrophoresis (SDS-PAGE) stained with Coomassie G-250. Protein concentrations were determined by the Bradford method using the Protein Assay Kit (Bio-Rad, Moscow, Russia) and bovine serum albumin as the standard. Molar concentration of enzyme solutions was determined by titration of the enzyme active sites with p′-guanidinobenzoic acid p-nitrophenyl ester as described in [28]. Buffer exchange was performed using a 30 kDa cutoff centrifugal filter device (Millipore, MA, USA).

To determine the oligomeric state of wild-type and modified PSP, the protein (2 mg/mL concentration) was applied to a Superdex 200 10/30 GL column (GE Healthcare, Chicago, IL, USA) equilibrated with 20 mM Tris-HCl, pH 8.0 and 200 mM NaCl.

### 2.3. Enzymatic Study

Kinetic parameters of substrate hydrolysis by wild-type and modified PSP variants were determined as described in [28,29]. Briefly, hydrolysis of Nα-benzoyl-D,L-arginine-p-nitroanilide (BAPNA) (Sigma-Aldrich, St. Louis, MI, USA) and two other p-nitroanilide (pNA) substrates, Z-RR-pNA and Z-KR-pNA (Z = benzyloxycarbonyl) (Bachem AG, Budendorf, Switzerland), was monitored as an increase in the absorption at 405 nm (25 °C) due to the formation of free p-nitroaniline (Δε405 = 10.400 M^−1^ × cm^−1^).

The initial hydrolysis rates were determined from the initial linear part of the kinetic curve (extent of hydrolysis did not exceed 10%) by monitoring the increase in the absorbance at 405 nm in 0.1 M Tris-HCl, pH 8.0, 2% DMSO, at 25 °C. At least 10 concentration points (in duplicate or triplicate with different concentrations of the enzyme) of each substrate were used to determine kinetic constants, usually in the range of 0.02–0.4 mM. The variance of v/[E] values at identical substrate concentrations did not exceed 5–10%. Kinetic parameters (*Kcat* and *Km*) were calculated from the Michaelis–Menten equation using nonlinear regression. The standard error did not exceed 10%.

For evaluation of the effect of spermine on the initial hydrolysis rates, 14 nM of either PSP or PSPmod and 0.1 mM BAPNA were used. The reactions were carried out in triplicate for each concentration of spermine.

### 2.4. Far-UV Circular Dichroism Spectroscopy

CD spectra and absorption spectra of wild-type and modified PSP variants were recorded in wavelength range 180–320 nm on Chirascan spectrometer (Applied Photophysics, Leatherhead, Surrey, UK) with 1 nm slit width and 1 nm step at 20 °C. Shared-Access Equipment Centre “Industrial Biotechnology” of Federal Research Center “Fundamentals of Biotechnology” Russian Academy of Sciences provided the equipment. Protein samples (1 mg/mL) were prepared in a 10 mM Na-phosphate buffer, pH 8.0, supplemented with 40 mM NaF. Optical path length was 10 mm. Protein concentrations were verified using extinction coefficients of peptide bond at 205 nm. All measurements were repeated twice for each sample.

### 2.5. Differential Scanning Calorimetry

Protein samples (2 mg/mL) were prepared in a 25 mM Na-phosphate buffer, pH 7.83, in duplicate either supplemented or not with 2 mM spermine. The excess heat capacity of the denaturation was measured with DASM-4M differential adiabatic scanning microcalorimeter with 467 μL capillary cells. The experiment was performed under a constant pressure of 2.2 atm at a heating rate of 1 K/min.

### 2.6. Protein Crystallization, Data Collection, Processing, Structure Refinement and Analysis

Crystallization of oligopeptidase B from *S. proteomaculans* with modified hinge region and its E125A and S532A mutants are described in [34,35]. Diffraction data from the crystals were collected at the Kurchatov synchrotron (beamline “BELOK”) and processed as described in [34,35]. The structures were solved by the molecular replacement method using BALBES program [36]. The refinements of all structures were carried out using the REFMAC5 program of the CCP4 suite [37]. The visual inspection of electron density maps and the manual rebuilding of the model were carried out using the COOT interactive graphics program [38]. In all final models, an asymmetric unit contained one independent copy of the protein. The visual inspection of the structure was carried out using the COOT program [38] and the PyMOL Molecular Graphics System, Version 1.9.0.0 (Schrödinger, New York, NY, USA). Contacts and free solvation energy of interdomain interface were analyzed using PDBePISA [39]. The structural comparison and superposition were made using the LSQKAB program [40]. The closest structural homologues were found and compared using the DALI program [41].

Data collection and refinement statistics are shown in Table 1. The real-space correlation coefficient plots for 7OB1, 7NE4 and 7NE5 PDB entries obtained using OVERLAPMAP software from the CCP4 suite are shown in Supplementary Figure S1.

### 2.7. Data Bank Accession Numbers

The structures of oligopeptidase B from *S. proteomaculans* with modified hinge region and its E125A and S532A mutants have been deposited to the Protein Data Bank (PDB) under accession codes (ID) 7OB1, 7NE4, 7NE5, respectively.

### 2.8. SAXS Measurement

SAXS experiments were carried out at the BM29 beamline at the ESRF (Grenoble, France) using a PILATUS3 2M 0n-vac (DECTRIS, Baden, Switzerland). Protein samples were prepared at three different protein concentrations (4.5, 9 and 18 mg/mL) in 20 mM TrisHCl buffer, pH 8.0, and 100 mM NaCl and were measured at 20 °C. The sample delivery and measurements were performed using a 1 mm diameter quartz capillary, which is a part of BioSAXS automated sample changer unit. Before and after each sample measurement, the corresponding buffer was measured and averaged. All experiments were conducted with following parameters: beam current—200 mA, flux—2.6 × 10^12^ photons/sec, wavelength—1 A, estimated beam size—1 mm × 100 um. A total of 10 frames (1 frame per second) were taken from each sample.

Data analysis software ATSAS 3.0.3 [42] and BioXTAS RAW [43] were used for the processing of SAXS profiles. The radius of gyration (Rg) and total forward scattering at zero angle (I(0)) were calculated using the Guinier approximation.

Pair-distance distribution function (PDDF) and maximum particle dimension (Dmax) were computed using GNOM [44]. Globularity and flexibility of the proteins were analyzed by using dimensionless Kratky volume-of-correlation (Vc)-based plot [45]. Low-resolution bead (dummy atom) model reconstructions of protein shape from SAXS data were generated in DAMMIN [46] using slow regime. The visualization of the bead model, derived density maps and the subsequent fitting of full-atomic models were performed using UCSF Chimera [47]. Theoretical scattering curves for the crystal structure (pdb id: 7ob1) and for the homology models of open and closed forms were calculated and compared with experimental curves using FOXS [48] and CRYSOL [49].

## 3. Results and Discussion

### 3.1. Comparative Analysis of Physicochemical Features and Enzymatic Activity of PSPmod

The peptidases of the POP family, including OpB, contain two domains—β-propeller and catalytic domains—linked through two hinge peptides (Supplementary Figure S2). The first peptide follows the N-terminal part of the catalytic domain (N-terminal loop) and links it with the β-propeller domain. The second follows the β-propeller and links it to the C-terminal part of the catalytic domain—α/β-hydrolase tertiary fold. Earlier, we showed that the chymotrypsinolysis of PSP in native conditions leads to the appearance of a 66 kDa enzyme with a truncated N-terminal loop, which retains activity towards low-molecular weight substrates and is also active towards a high-molecular weight substrate, azocasein [50]. This product of limited proteolysis was not stable and suffered from further degradation. The production of truncated PSP, in which the N-terminal loop was deleted by site-directed mutagenesis, in *E. coli* resulted in the expression of insoluble proteins with undetectable catalytic activity after refolding (data not shown). Finally, the tobacco etch virus (TEV) protease digestion site (ENLYFQ*S) was introduced by site-directed mutagenesis instead of amino acids 71-77 (IPQQEHS), forming the first hinge peptide (Supplementary Figure S2).

The recombinant protein with a modified hinge region (PSPmod) was expressed in *E. coli* and purified to homogeneity. The TEV recognition site was confirmed by Maldi-TOF mass spectrometry but was not susceptible to TEV-protease hydrolysis. This low accessibility for TEV-protease was supposedly caused by strong interactions of the peptide with neighboring protein regions.

**Figure 1:**
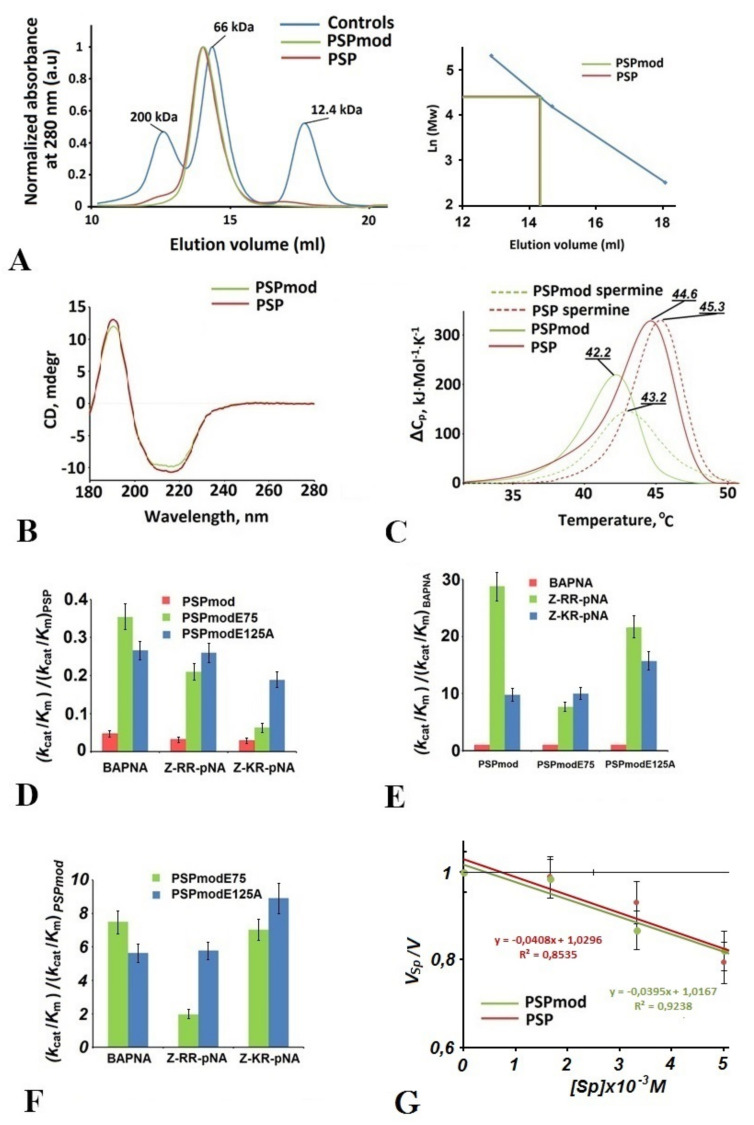
Comparative analysis of physicochemical features and enzymatic activity of PSPmod. (**A**) SEC was performed on the Superdex 200 10/300 GL column. Proteins (2 mg/mL) were eluted in 20 mM Tris-HCl (pH 7.8) and 100 mM NaCl buffer; (**B**) CD spectra normalized to the optical density value (0.5 at 205 nm). Absorption spectra were measured simultaneously with CD. The means of 3 measurements are presented; (**C**) temperature dependence of the excess heat capacity of denaturation (ΔCp) measured by DSC for PSP and PSPmod in the presence and absence of spermine. In all cases, 2.0 mg/mL of protein was dissolved in 25 mM Na-phosphate buffer (pH 7.8) supplemented with 2mM spermine, when indicated. Temperatures of melting curve maxima (Tmax) are shown at the top of the peaks; (**D**–**F**) kinetic parameters of hydrolysis (kcat/Km) normalized as indicated in the *Y*-axis legend. The corresponding parameters for wild-type PSP (**D**) and PSPmod (**F**) toward the indicated substrates are equal to 1 and not shown; (**G**) the effect of spermine on the initial rates of hydrolysis of BAPNA by PSP and PSPmod.

To ensure that PSPmod does not possess major distortions in the oligomeric state or secondary structure composition compared to wild-type PSP, the proteins were evaluated by size-exclusion chromatography (SEC) and circular dichroism spectroscopy (CD), respectively, while their thermal stabilities were measured by differential scanning calorimetry (DSC) (Figure 1A–C).

According to SEC, both proteins were eluted as monomers with molecular weights of 80 kDa (calculated MW is 76 kDa) (Figure 1A). This corresponds to the previously obtained data that found that monomers are functional forms of γ-proteobacterial OpB [30,31,32]. In contrast, protozoan OpB, including TbOpB and LmOpB, were proven active dimers in solution [26,27].

Analogously, the CD spectra of PSP and PSPmod were close to each other, excluding minor differences (Figure 1B).

The thermal denaturation of PSP and PSPmod was studied by DSC at a protein concentration of 2.0 mg/mL in a 25 mM sodium phosphate buffer (pH 7.8). The corresponding melting curves presented in Figure 1C show the downshift by 2 °C of position of the melting curve maximum (Tmax) of PSPmod compared to PSP, whose Tmax at 44.6 °C was well correlated with previously studied thermal inactivation of the enzyme [51]. Incubation at 43 °C caused the reversible denaturation of PSP, while heating to 46 °C led to irreversible activity loss.

The enzymatic activity of PSPmod was measured and hydrolysis efficiency (*k_cat_/K_m_*) was compared with that of wild-type PSP. Three p-nitroanilide substrates were used: BAPNA, the substrate lacking a residue in the P2-position and interacting only with the S1 substrate-binding site, and substrates Z-(R/K)R-pNA, with different basic residues in the P2 position.

Table 2 and Figure 1D show that PSPmod displays low activities towards all substrates, constituting from 3 to 5% from those of PSP. At the same time, such characteristics of substrate specificity of PSP as enhanced activity towards dibasic substrates or preference to the Arg residue over Lys in the P2 position have not been changed (Figure 1E).

The hinge region modification included the substitution of the functionally important residue Glu75, which, according to our previous homology modeling and molecular dynamics studies, participated in the putative interdomain SB network stabilizing PSP in the closed conformation [28,29]. The alanine substitution of Glu75 caused a loss in activity of wild-type PSP toward three p-Na substrates, from 82 to 92% [28]. The reinstallation of Glu75 residue in PSPmod by additional site-directed mutagenesis increased the hydrolysis efficiencies of PSPmodE75 toward BAPNA, Z-RR-pNA, and Z-KR-pNA over those of PSPmod by about 7-, 2- and 7-fold, respectively (Figure 1F), which corresponds to the restoration of activities towards corresponding substrates to 35, 21 and 6% compared to wild-type PSP (Figure 1D).

The partial restoration of the catalytic activity of PSPmod was also achieved by the alanine substitution of Glu125 acidic residue from the β-propeller domain: PSPmodE125A possesses hydrolysis efficiency toward the substrates, from 19 to 26%, of PSP (Figure 1D), demonstrating increases in hydrolysis efficiency (*k_cat_/K_m_*) over PSPmod by about 6- to 9-fold (Figure 1F). According to our previous in silico modelling, Glu125 also participated in the putative interdomain SB network and its alanine substitution increased the activities of PSP toward BAPNA and dibasic substrates by about 8- and about 2-fold, respectively [28,29].

Previously, using differential scanning fluorimetry (Thermofluor), we found that low-molecular weight polyamines, e.g., spermine (Sp), could stabilize PSP in solution [34]. Here, we evaluated the Sp influence on thermal denaturation and catalytic activity of PSP and PSPmod. DSC showed that the polyamine causes one degree increases in Tmax for both proteins (Figure 1C). This finding is correlated with known chaperone and stabilizing effects of spermine on serine proteases [52]. The effect of Sp on the catalytic activity of both PSP and PSPmod was similar: 5 mM Sp caused 20% inhibition of the initial rate of hydrolysis (Figure 1G). Despite its small effect on thermal stability, the presence of Sp made it possible to obtain crystals suitable for X-ray analysis for PSPmod and its derivatives, PSPmodE125A and PSPmodS532A [35]. PSPmodS532A carried the alanine substitution of the catalytic triad serine and did not possess any enzymatic activity. Neither wild-type PSP nor its corresponding mutated variants were crystallized, indicating that the combination of both the hinge region modification and spermine presence is required for crystallization.

### 3.2. Intermediate States Were Observed in the Crystal Structures of PSPmod and Its Derivatives

#### 3.2.1. Structural Overview

**Figure 2:**
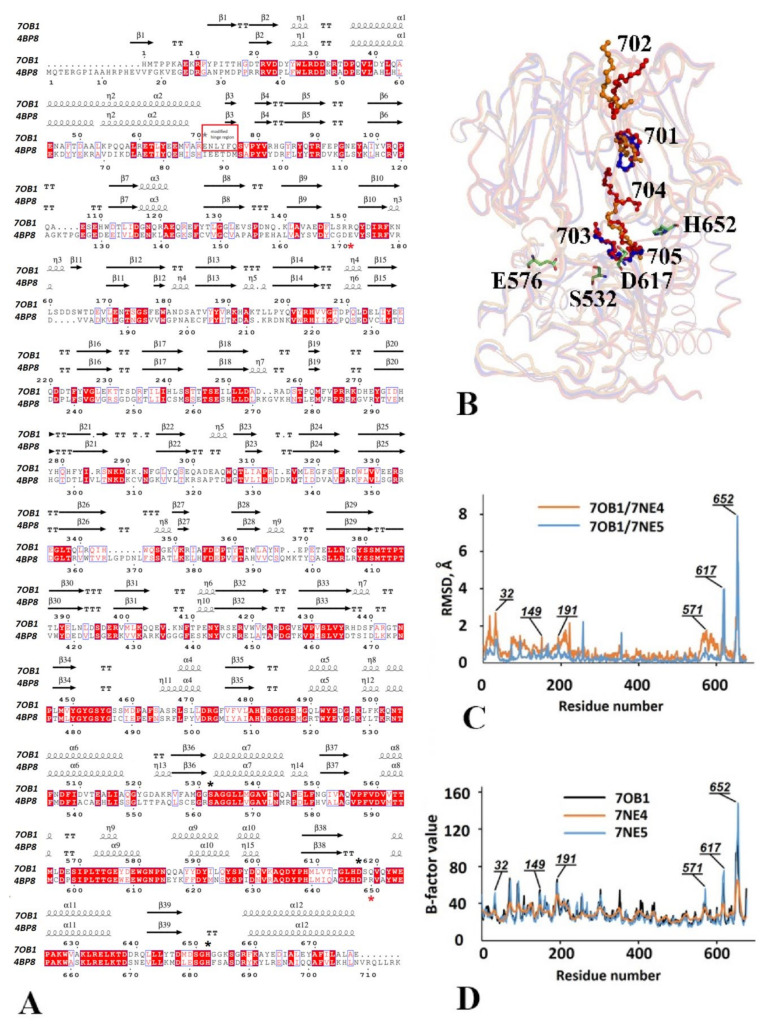
Overview of the crystal structures of PSPmod and its derivatives. (**A**) Multiple sequence and structural alignment of PSPmod (7OB1) and TbOpB (4BP8). The alignment was generated with ESPript (http://espript.ibcp.fr; accessed on 5 September 2021). Highly conserved residues are highlighted in red; semi-conserved ones are colored red. Catalytic triad and S1 substrate-binding site residues are marked with black asterisks; the interdomain salt bridge SB1 of TbOpB is marked with red asterisks; the modified hinge region is in red squire. Secondary structure elements are shown above the alignment. (**B**) Superposition of PSPmod (PDB ID 7OB1, in red), PSPmodE125A (PDB ID 7NE4, in orange) PSPmodS532A (PDB ID 7NE5, in blue) with spermines in the interdomains cavities. The spermine molecules are shown in ball and sticks and numbered according to the PSPmod structure (PDB 7OB1). The catalytic triad and S1 substrate-binding center residues of PSPmod are shown as green sticks. (**C**) Distributions of RMSD values along the PSP sequence. RMSD values were calculated upon Ca-atom superpositions of PSPmodE125A (PDB ID 7NE4) and PSPmodS532A (PDB ID 7NE5) over PSPmod (PDB 7OB1). (**D**) Distributions of the residual B-factors of the three crystal structures along the PSP sequence. Residues with the highest values of both RMSD and B-factor are shown in (**C**,**D**).

The three-dimensional structures of PSPmod (PDB ID 7OB1) and its derivatives, PSPmodE125A (PDB ID 7NE4) and PSPmodS532A (PDB ID 7NE5), were determined at 2, 2.72 and 1.88 Å resolutions, respectively (Table 1). In all structures, polypeptide chains were folded similarly to TbOpB, forming the α/β-hydrolase and β-propeller domains connected through the hinge region (Figure 2A,B). The polypeptide chain contains 685 amino acid residues, including nine residues of the N-terminal His-tag (MASHHHHHH) undetectable in electron densities, except for the last His in the PSPmod structure. The Cα-atom superposition of the structures 7NE4 and 7NE5 on 7OB1 results in RMSD values of 0.9 and 0.6 Å, indicating the practical identity of folding of PSPmodE125A and PSPmodS532A compared to PSPmod (Supplementary Table S1). The superimposition of structures and the variation of RMSD values along the polypeptide chains are presented in Supplementary Figure S3 and Figure 2C, respectively. The figures show that variations of folding are mostly associated with flexible loops of the β-propeller and catalytic domains, where, by using B-factor analysis, enhanced intrinsic flexibilities of polypeptide chains were observed (Figure 2D).

The crystals of PSPmod and its derivatives have been grown in the presence of 5 mM Sp in the crystallization solution. As a result, five, three and two bound Sp molecules were found in the structures of PSPmod, PSPmodE125A and PSPmodS532A, respectively (Figure 2B). In PSPmod, Sp702 is situated on the surface of the propeller domain near the η4-helix. Three Sp line the surface of the interdomain cavity of the catalytic (Sp703 and Sp705) and β-propeller (Sp701) domains, and Sp704 is located between the domains. Sp703 is remarkably close to catalytic Ser532: the distance 703C11-Ser532OG is 4.11 Å. Sp701 is present in all structures, while Sp704—only in the PSPmod structure. The second Sp of the PSPmodS532A structure is in the proximity of catalytic Ser532 similarly to Sp703 in the PSP structure. The second and third Sp of PSPmodE125A occupy the positions of Sp702 and 705 in PSPmod, respectively.

The catalytic domain consists of a short N-terminal loop (residues 1–70) and a long C-terminal α/β-hydrolase fold (residues 411–676). The β-propeller domain (residues 77–404) is inserted between these two regions of the catalytic domain and links with them covalently through two linear peptide strands, containing residues 71–76 and 405–410, respectively (the hinge) (Figure 2A). In PSPmod and its derivatives, the amino acid sequences of the first hinge peptide were completely modified compared to wild-type PSP (see previous section). B-factor analysis showed an enhanced flexibility of this area compared to the second hinge peptide (Supplementary Figure S3).

**Figure 3:**
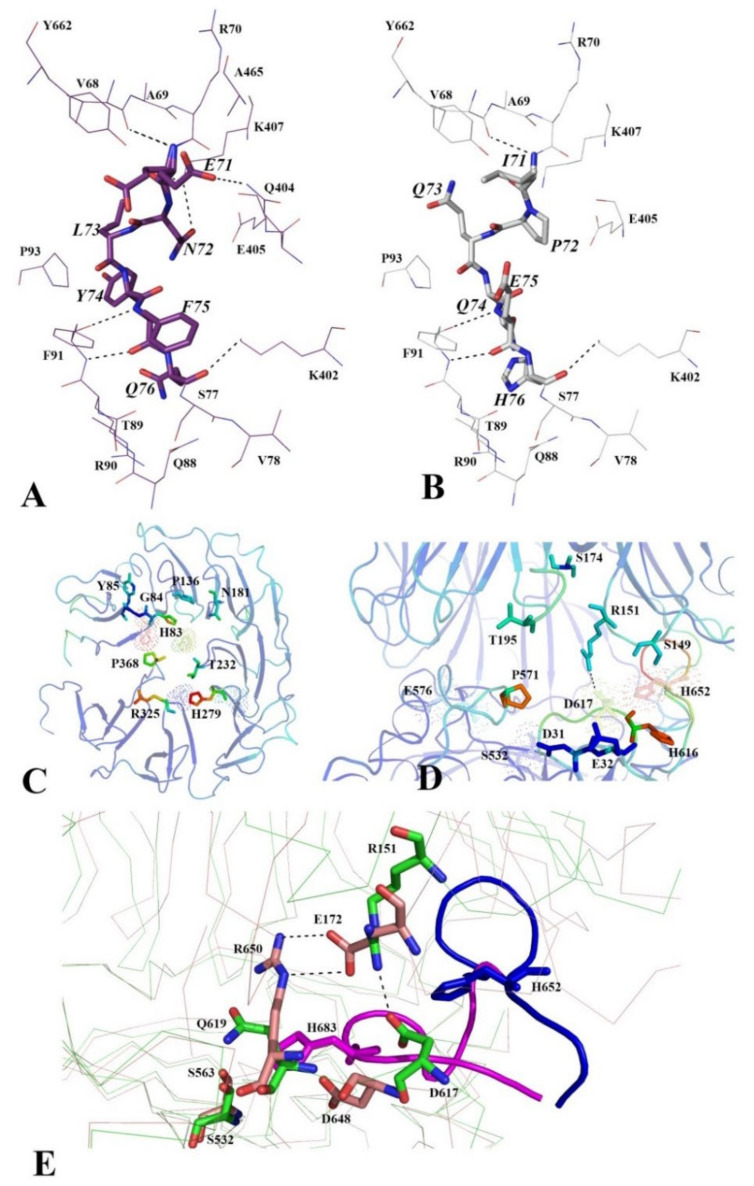
The modified hinge region, access to the interdomain cavity, and the configuration of the catalytic triad residues in the spatial structure of PSPmod. (**A**) The amino acid environment and polar contacts of the modified hinge region (shown in magenta). (**B**) Expected changes during the return of the original sequence of the hinge region (shown in grey). (**C**) A cartoon image of the entrance to the channel, which connects the upper part of the β-propeller domain with the cavity between the domains, and the amino acid residues, which limit the perimeter of the channel. The color change from blue to red reflects the shift of the B-factors from low to high. (**D**) A similar representation of the opening in the inter-domain interface and the amino acid residues that restrict the entrance to the opening. The catalytic triad residues and S1 substrate-binding center are represented by translucent dotted images. A dotted line shows the interaction between Arg151 and the catalytic Ser532. (**E**) Comparison of the configurations of the catalytic triads and salt bridges involved in the stabilization of the catalytic Asp positions in PSPmod (green) and closed state of TbOpB (beige). The His-loops are colored blue and purple for PSPmod and TbOpB, respectively. Amino acid residues are shown with sticks; salt bridges discussed in the text are marked with dotted lines.

An analysis of intramolecular interactions involving the modified hinge revealed that it has multiple contacts, mostly with the second hinge strand and the neighboring parts of the catalytic and β-propeller domains, including polar contacts with residues Val68 from the α2-helix of the N-terminal loop, Glu405 and Lys 407 of the second hinge, and Phe92 and Lys402 from the β5- and β31-strands of the β-propeller domain (Figure 3A). A similar analysis performed after the reinstallation of the native sequence in the modified region shows a preservation of the interactions with the catalytic and β-propeller domains, while polar contacts with the second hinge peptide were lost (Figure 3B). A comparison of the modified (ENLYFQ) and original (IPQQEH) sequences of the hinge peptide showed that the overal composition of charged/polar and aliphatic amino acids was identical, but their local orders were different and the charged N-terminal part of modified hinge led to the formation of the additional polar interactions shown in Figure 3A.

The α/β-hydrolase fold contains a central core from the twisted β-sheet consisting of eight β-strands surrounded by eight α-helices and seven short η-helices (Figure 2A). Only the last two β-strands of the twisted sheet are in an antiparallel orientation, whereas the others adopt a parallel conformation. The N-terminal loop consists of a two-stranded antiparallel β-sheet and two α-helices with one η-helix between them. The segment wraps around the surface of the domain adjacent to the long C-terminal α-helix. The surfaces of the catalytic and propeller domains facing each other participate together in the creation of an interdomain cavity, where catalytic and substrate binding sites are located (Figure 2B).

The β-propeller domain (residues 77–404) is built out of seven blades, each of which is composed of four antiparallel β-strands (Figure 2A). These blades form a central barrel with a narrow channel inside which passes between the propeller blades and connects the top of the domain surface to the interdomain cavity (Figure 3C). The perimeter of the channel is restricted by the amino acid residues of flexible loops joining pairs of β-strands: His83-85 between β4 and β5; Pro368 between β28 and β29; Pro136 between β8 and β9; Asn181 between β12 and β13, Thr232 between β16 and β17; His279 between β20 and β21; Arg325 between β24 and β25. The distances between Ca-atoms of amino acid residues on opposite sides of the channel are 12.84, 11.68 and 12.77 Å for Pro368-Pro136, Asp234-His83, Arg82-Asp234, respectively. Corresponding distances in the narrowest part are almost half as wide.

In addition to the channel, access of the solution, small molecules, and peptides into the interdomain cavity is possible through an opening between flexible loops linking the blade strands of the propeller with some of the α-helices and β-strands of the catalytic domain along the perimeter of the interface (Figure 3D). The opening is restricted by residues Asp31 and Glu32 (η1), Ser174 (β12), His616 (α10), Ser149 (β9-β10 loop), Pro571 (α8-α9 loop), and Thr 195 (β13-β14 loop). The distances between Cα-atoms of amino acid residues which defined the size of the opening are 10.1, 16.5 and 7.7 Å for Ser149-His616, Ser174-Pro571 and Thr195-Pro571, respectively.

The side chains of Arg151 from the β-propeller and catalytic Asp617 bond together and form a salt bridge (Asp617OD–Arg151NH2 distance is 2.75 Å, while a distance of 10.5 Å is between Cα-atoms), which blocks the entrance into the interdomain cavity through the opening (Figure 3D).

#### 3.2.2. The Catalytic Triad Arrangement

The catalytic triad of PSP, which creates a charge-relay system for a nucleophilic attack by the catalytic Ser during hydrolysis, consists of Ser532, Asp617, His652 amino acid residues (Figure 2A,B). Ser532 is located inside the interdomain cavity, on the tip of the sharp turn between strand β36 and helix α7; its side chain faces the propeller domain. Asp617 is located closer to the enzyme surface, on the flexible loop (residues 615–623) between strand β38 and the α11-helix. The third residue of the catalytic triad, His652, is situated in the very flexible long His-loop (residues 648–658) between strand β39 and the α12 C-terminal helix. The majority of amino acid residues of the His-loop have the highest B-factor values in the PSPmod structure (Figure 2D and Supplementary Figure S3). Poor electron densities in His-loop areas are typical for spatial structures of ligand-free bacterial and fungal PEP crystallized in the open states (Table 3). Table 3 shows that in a structure of ligand-free TbOpB, where the His-loop is well defined [26], distances between catalytic residues involved in nucleophilic attacks are significantly longer than those in the closed state. The shift of the Cα-atom of catalytic His during the TbOpB transition between two conformations reaches 10Å (Table 3). In the PSPmod structure, the distances between Cα-atoms in the pairs Ser532–His652 and His652–Asp617 are equal to 18.2 and 10.6 Å, respectively, which are longer than those in the closed states of TbOpB and ApPEP and comparable with those in the open state of TbOpB and intermediate states of PfPEP and GmPEP (Table 3). Similar distances are observed in the structures of PSPmod derivatives (Supplementary Table S1).

In PSPmod, the His-loop location outside of the active center is stabilized by hydrophobic contacts of Met648 with Leu42, Glu43 and Asp46 residues of the α1-helix from the N-terminal loop.

#### 3.2.3. Comparative Analysis of the Domain Positioning

In POP family enzymes, domains arrangement in the open and closed states can be differentiated using several parameters characterizing the degree of the domain’s closeness/separation, e.g., the distance between centers of mass of the domains, percentage of buried (interface) area from the total area, numbers of residues in the interdomain interface and their interactions between each other.

In order to conduct the comparative analysis of the domain arrangement in PSPmod, the search for homologous assemblies and their primary evaluation were performed using Dali online service [41]. The analysis revealed several POP family representatives, which were crystallized in different conformations. A comparison of the structure of PSPmod with those of TbOpB, ApPEP, GmPEP and PfPEP is presented in Table 3. In contrast to the catalytic triad arrangement similar to that in TbOpB in the open state, the overall structures of modified PSP were better aligned with the closed states of TbOpB and ApPEP as well as the intermediate states of GmPEP and PfPEP.

According to Table 3, the distances between the centers of mass of the domains in the open states are 6–8 Å larger than in the closed (or intermediate) states. In contrast, the percentage of the buried surface area and amount of interface residues are 1.5–2 times less. A comparison of the aforementioned parameters indicates that PSPmod crystallized in the intermediate conformation, observed previously in the crystal structure of archaeal PfPEP [25] and catalytically impaired fungal macrocyclases GmPEP in complexes with macrocyclization substrates [24]. In the last case, the intermediate conformation, which demonstrated a combination of a disrupted state of the catalytic triad with closeness of the domains, was attributed to the consequences of the catalytic residue mutations.

In addition, we compared the domain arrangement in the structure of PSPmod with those of its derivatives, homologous models of wild-type PSP in different conformations (taken from [28]) and the model of EcOpB predicted by Alphafold [53] (https://alphafold.ebi.ac.uk/entry/P24555; accessed on 5 September 2021). According to Supplementary Table S1, two PSPmod derivatives adopted intermediate states. Interestingly, according to the RMSD values, the EcOpB model is very close to the PSPmod structure, but the distances between catalytic triad residues and domains arrangement clearly indicate the closed state of the enzyme. Assumingly, this reflects the fact that in the majority of the POP family-related structures from the PDB database, the enzymes are in the closed states. 

In all cases, when POP family enzymes were crystalized in the intermediate conformations, substrate-like compounds or substrates (in the case of mutated GmPEP) were presented in the interdomain cavities: prolylproline ligands in the PfPEP and spermine molecules in PSPmod. These ligands apparently contributed to the closure of domains, which, due to the lack of a substrate, was not associated with catalytic activation. Taking into account the presence of polyamines and other substrate-like molecules in bacterial (or archaeal) cells, spermine or prolylproline-induced (in case of PfPEP) conformational transition may replicate a naturally occurring stage of the enzyme functioning. A two-step catalytic activation representing the transition from an open state to a closed one through an intermediate state described here, in which domain closure precedes the formation of the working configuration of the catalytic triad, can be widely distributed in vivo.

A molecular dynamics (MD) study of PfPEP indicated that the intermediate conformation observed in the PfPEP crystal structures represents a transient state between much larger extremes, which can be reached by the enzyme, and suggested that the partial domains closure in the intermediate state does not completely prevent the catalytic His and Ser approach to a distance favorable for catalysis and a formation of the active site configuration analogous to those observed in the closed conformations of inhibitor-bound PEP [20]. The described openings above in the interdomain interface and in the top of the β-propeller allow substrate entrance to the active site of the intermediate state, though the sizes of the substrate would be restricted by the diameters of the openings.

#### 3.2.4. Functionally Important Interdomain Salt Bridge (SB1) Conserved in Protozoan OpB and Bacterial PEP Is Abscent in PSPmod

Snapshots of different conformational states obtained by a crystallographic study of bacterial and fungal PEP, and protozoan OpB, showed that the domains are able to move apart at an angle, opening like a book [12,13,26,27]. Synergy between catalytic activation and movement of the domains was suggested for protozoan OpB and bacterial PEP [26]. A key role of TbOpB in the proposed mechanism of catalytic activation was suggested for Glu172 occupying the position of Arg151 in PSP, which forms SB1 with Arg650 (Gln619 in PSP) in the closed conformation of TbOpB (Figure 3E). This SB1 keeps catalytic Asp648 (Asp617 in PSP) and His683 (His652 in PSP) in the positions favorable for catalysis. The transition to the open conformation (domains opening) caused a disruption of SB1 and as a result interaction of the free Arg650 with the neighboring catalytic Asp648. The interaction caused displacement of catalytic His683 from the proximity of catalytic Ser563 (Ser532 in PSP) and a consequent disruption of the catalytic triad [26]. The amino acid substitution of Glu172 caused significant loss of TbOpB catalytic activity [54].

In the obtained crystal structures of the intermediate state of PSPmod, the domains occupied positions similar to those observed in crystal structures of the closed form of TbOpB and related PEP. Gln619 was unable to form a SB with Arg151 and the latter interacted directly with catalytic Asp617 (Figure 3E), the interaction restricted His-loop movement and prevented rapprochement of His652 and Ser532 and consequent catalytic activation. Thus, it is possible to assume that the disruption of SB Arg151-Asp617 is rather favorable for catalysis. Neither alanine nor glutamate substitution of Arg151 caused significant PSP inhibition [29], which confirms that SB Arg151-Asp617 is not a functional analog of the TbOpB SB1, and the mechanism of catalytic activation proposed for protozoan OpB is not compatible with both the amino acid sequence of PSP and structural data presented here. Determination of the mechanism of catalytic activation of bacterial OpB require further experimental and/or computational studies, but prior their conduction we had to confirm that the intermediate state was not an artefact of crystallization and clarify its relation with both the hinge modification and spermine presence.

### 3.3. SAXS Analysis of the Conformation of PSP and Its Derivatives in Solution

The first structure of bacterial OpB was obtained for PSPmod—an enzyme with a modified hinge region and in the presence of spermine, whose molecules were accumulated in the interdomain cavity. Either one of these factors, or their combination, could promote a stabilization of PSP in the intermediate state. To shed light on the conformational state of PSP and its derivatives in solution, we performed SAXS measurements.

**Figure 4:**
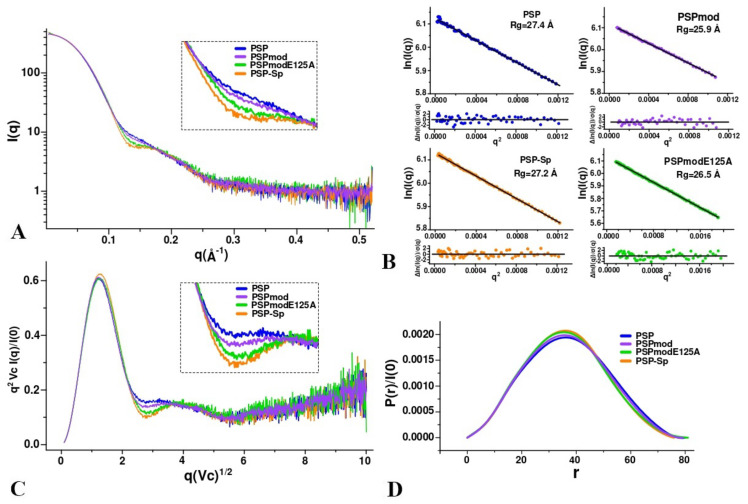
Analysis of SAXS data for various PSP derivatives. The experimental conditions are the same for all measurements (20 mM TrisHCl buffer, pH 8.0 and 100 mM NaCl, T = 20 °C). (**A**) SAXS curves on a logarithmic scale (the inset shows the region with the highest deviation); (**B**) Guinier plot with linear fit; (**C**) dimensionless (normalized) volume-of-correlation(Vc)-based Kratky plots; (**D**) pair-distance distribution function profiles (GNOM).

SAXS data were obtained for PSP, PSP in the presence of spermine (PSP-Sp), PSPmod and PSPmodE125A (Figure 4). In order to exclude the influence of interparticle interaction and aggregation on the SAXS profiles, measurements at different concentrations were performed. Data obtained at a protein concentration of 4.5 mg/mL were selected, since there is no deviation of Ln(I) at low q from the linear dependence in the Guinier plot (Figure 4B). Rg and I(0) were determined for all profiles using Guinier’s approximation (Table 4). These results support the monomeric state of all PSP derivatives in the aqueous solution.

The analysis of SAXS profiles in dimensionless (Vc-based) Kratky coordinates allows us to determine the degree of order and flexibility of the protein. In all cases, the profiles corresponded to a globular protein with an “implicit” multi-domain form (Figure 4C), since there was a minor peak in addition to the major. The behavior of the profiles in the region between peaks (inset in Figure 4C) suggests that the degree of conformational flexibility decreases in the order: PSP, PSPmod, PSPmodE125A, PSP-Sp. PDDF profiles have a Gaussian-like shape with a main peak at 36 Å (Figure 4B), which corresponds to a structured globular protein. The maximum protein size (Dmax) according to PDDF (Table 4) for PSP-Sp corresponds to the lowest value in comparison with other forms. This indicates that some degree of globule compaction occurs when spermine binds to PSP. The PDDF profile of PSP slightly broadens towards increasing distance. This behavior may indicate a higher cavity volume of PSP compared to PSPmod. The PDDF profile of PSPmod has an intermediate width and reaches the minimum value for PSPmodE125A and PSP-Sp. This is consistent with the minimum distance between the center of mass of the domains and the maximum value of the buried surface area found in the crystal structure of PSPmodE125A compared to PSPmod (Supplementary Table S1).

**Figure 5:**
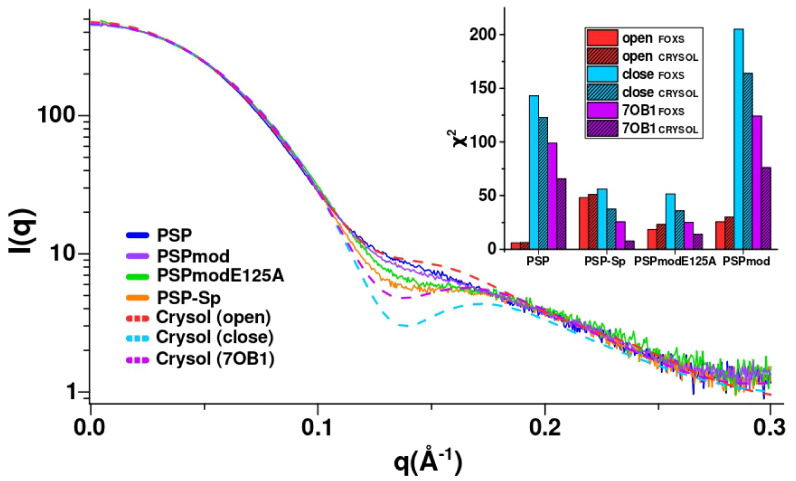
Experimental SAXS profiles (solid) and theoretical (dashed) calculated using CRYSOL for homologous PSP models in open and closed conformations and crystal structure (PDB ID: 7OB1). The inset shows the histogram of the chi-square distribution for FOXS/CRYSOL calculations.

Further, the experimental curves were compared with theoretical curves calculated for the PSPmod crystal structure (7OB1) and homologous PSP models in the open and closed conformations obtained earlier [28]. The calculations were performed twice using both FOXS and CRYSOL programs. The best fit was observed between the curves for PSP and the modelled open conformation, as well as for PSP-Sp and 7OB1 crystal structure (Figure 5 and Table 5).

The results obtained indicate that a closed conformation (similar to those found in the crystal of inhibitor-bound protozoan OpB and bacterial PEP) does not exist in the solution, since the theoretical curve for the closed form does not match any experimental scattering profile. We can assume that spermine-free PSP exists in an open conformation or in its dynamic equilibrium with a small fraction of an intermediate conformation observed in the crystal structure of PSPmod. Upon spermine binding, a conformational transition of PSP to the intermediate state resembling those in 7OB1 occurs. The SAXS profile for PSPmod is in good agreement with the linear combination of the experimental profiles of PSP and PSP-Sp in a 7 to 3 ratio, which indicates that PSPmod has significantly higher content of the intermediate state fraction compared to PSP. Analogously, if the differences in the SAXS profiles are determined by the ratio of the intermediate and open conformation in the solution, then the intermediate conformation dominates for PSPmodE125A.

**Figure 6:**
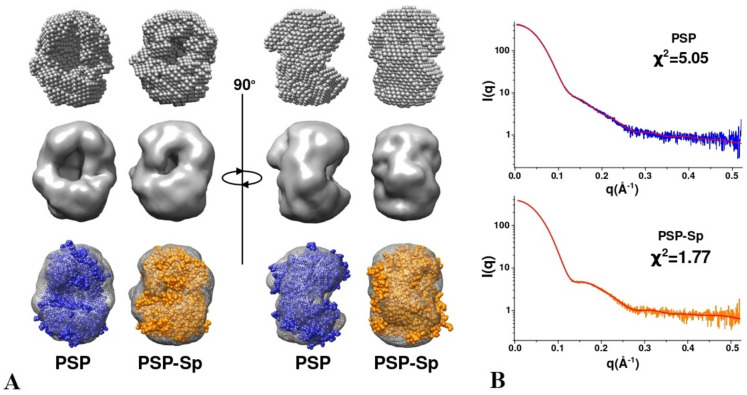
Ab initio shape reconstruction for PSP and PSP-Sp using DAMMIN. (**A**) Bead models, density maps with 12 Å resolution and full-atom models of open PSP state (blue) and 7OB1 (orange) fitted in them; (**B**) comparison of the experimental SAXS profiles of PSP and PSP-Sp with the corresponding theoretical profiles of DAMMIN ab initio models (red line).

To visualize the detected difference between PSP and PSP-Sp, we have performed ab initio shape determination by simulated annealing using DAMMIN [46] (Figure 6). The resulting bead models of PSP and PSP-Sp were transformed to a density map with 12 Å resolution, then full-atom homologous models of the open and intermediate state of PSP were fitted into the density maps of PSP and PSP-Sp, respectively (Figure 6A). The number of beads for PSP and PSP-Sp after simulated annealing was 2138 and 2462, respectively. This fact and the results of fitting indicate an open state of PSP. The large surface-exposed cavity in the ab initio PSP model corresponds to the cavity formed during the relative reorientation of the two domains in the ligand-free state (Figure 6A).

SAXS data obtained for PSP and its derivatives suggested that in solution wild-type PSP exists in the open conformation. Upon spermine binding, a domain closure and transition to the intermediate conformation occurs. Due to the substrate absence, the process is not associated with formation of an active configuration of the catalytic triad. A conformation of PSPmod in solution supposed to be close to PSP except for suggested presence of both open and intermediate conformations in dynamic equilibrium. We can suggest that the increase in the initial (spermine-independent) intermediate conformation can favorably affect the nucleation process by increasing the effective protein concentration since the intermediate state forms the crystalline phase.

## 4. Conclusions

In this study, we described, for the first time, a crystal structure of bacterial oligopeptidase B from *Serratia proteomaculans* (PSP)—a two-domain, trypsin-like enzyme from prolyloligopeptidase (POP) family. The structure was obtained for an enzyme with a modified hinge region (PSPmod) and in the presence of spermine. The activity loss caused by the modification was partially reversed by either a reinstallation of functionally important Glu75 in PSPmod or additional alanine substitution in the interdomain interface (Glu125Ala). In the same time, oligomeric states, secondary structure compositions and thermodynamic features of PSP and PSPmod were identical and similar, respectively, indicating that the obtained structural data are applicable for the elucidation of the mechanism of catalytic activation of bacterial OpB and its comparison with those suggested for protozoan OpB and other representatives of POP family.

PSPmod and two its derivatives (PSPmodE125A and PSPmodS532A) were crystallized in intermediate conformations, which are characterized by a disruption of the catalytic triad typical for ligand-free enzymes in open states, while domains’ closeness resembled closed states of ligand-bound POP. Neither wild-type PSP nor its corresponding mutated variants were susceptible to crystallization, indicating that the hinge region modification was promoting crystal growth. The influences of the hinge region modification and spermine on the conformational state of PSP in solution were evaluated by small-angle X-ray scattering.

SAXS showed that, in solution, wild-type PSP exists in the open state, while spermine caused the transition to the intermediate state observed in the PSPmod crystal structure. PSPmod was similar to PSP to a certain extent: the difference in the SAXS profiles can be attributed to the substantial fraction of the intermediate state. These findings confirm that both hinge region modifications and substrate-like ligands affect conformational state of PSP.

We suggest that spermine-dependent conformational transition of PSP replicates the behavior of OpB in bacterial cells. Similarly to spermine, other small-molecule compounds could cause a transition from the open to intermediate state. The openings in the interdomain interface and/or in the top of a β-propeller allow small substrates to enter to the interdomain cavity of the intermediate state. Binding of the substrate causes catalytic activation—a transition from the intermediate to closed state. This two-step catalytic activation, when domain closure precedes the formation of the working configuration of the catalytic triad, might coexist with classical catalytic activation associated with the transition from the open to closed state described for protozoan OpB and bacterial PEP.

Thus, structural data presented here complement our knowledge on the diversity of sequence–structure–catalytic activation relationships in the POP family and indicate that the intermediate state should be considered during computational studies, including those aiming to develop the small-molecule inhibitors targeting corresponding enzymes. Since the composition of cytoplasm is significantly richer than buffer/salt solution commonly used in biochemical and structural studies, the intermediate state, which is rarely detected in vitro, might be widely distributed in vivo.

## Figures and Tables

**Table 1:** Data collection, processing, and refinement.

PDB ID Proteins	7OB1 PSPmod	7NE5 PSPmodS532A	7NE4 PSPmodE12A
**Data collection**			
Diffraction source	K4.4 beamline, NRC “Kurchatov Institute”	K4.4 beamline, NRC “Kurchatov Institute”	K4.4 beamline, NRC “Kurchatov Institute”
Wavelength (Å)	0.79272	0.79272	0.79272
Temperature (K)	100	100	100
Detector	CCD	CCD	CCD
Space group	P2_1_2_1_2_1_	P2_1_2_1_2_1_	P2_1_2_1_2_1_
*a*, *b*, *c* (Å)	73.21; 101.02; 108.89	70.71, 100.40, 108.67	68.84, 98.56, 108.26
α, β, γ (°)	90.0	90.0	90.0
Unique reflections	55364 (3999)	63282 (4622)	20453 (1476)
Resolution range (Å)	20.0–2.00 (2.10–2.00)	47.8–1.88 (1.93–1.88)	44.9–2.72 (2.79–2.72)
Completeness (%)	99.90 (99.89)	99.80 (99.78)	99.92 (99.86)
Average redundancy	7.84 (4.22)	7.25 (4.31)	6.18 (5.96)
〈I/σ(I)〉	23.3 (5.45)	10.15 (2.09)	8.45 (2.11)
Rmrgd-F (%)	5.2 (26)	4.9 (31)	6.1 (29)
**Refinement**			
*R_fact_ (%)*	20.8	20.9	25.2
*R*_free._ *(%)*	24.9	25.2	30.5
Bonds (Å)	0.01	0.01	0.004
Angles (°)	1.63	1.63	1.02
*Ramachandran plot*			
Most favoured (%)	99.2	99.2	99.2
Allowed (%)	0.8	0.8	0.8
*No. atoms*			
Protein	5545	5534	5531
Water	216	386	50
Ligands	70	28	42
B-factor (Å2)	28.432	29.393	28.828

Values in parenthesis are for the highest-resolution shell.

**Table 2:** Kinetic parameters of hydrolysis of three substrates by PSPmod and its derivatives (0.1 M Tris-HCl, pH 8.0; 2% DMSO, 25 °C). The standard error did not exceed 10%.

Enzyme	BAPNA			Z-RR-pNA			Z-KR-pNA		
	*k_cat_* min^−1^	*K_m_* µM	*k_cat_*/*K_m_* × 10^−7^ M^−1^ min^−1^	*k_cat_* min^−1^	*K_m_* µM	*k_cat_*/*K_m_* × 10^−7^ M^−1^ min^−1^	*k_cat_* min^−1^	*K_m_* µM	*k*_cat_/*K_m_* × 10^−7^ M^−1^min^−1^
PSPmod	91.7	188.8	0.049	157.3	11.18	1.41	592.8	124.4	0.477
PSPmod E75	603.1	165.5	0.364	960	34.65	2.77	805.7	24.0	3.36
PSPmod E125A	728.7	265.6	0.274	424.0	5.92	8.15	493.3	11.6	4.25
PSP	791.2	78.1	1.01	2181.2	4.35	50.1	3045.6	20.8	14.6

**Table 3:** Catalytic triad and domains positioning in the crystal structure of PSPmod and those of TbOpB, ApPEP, GmPEP and PfPEP crystallized in different conformational states.

PDB ID	7OB1	4BP8	4BP9	3IUL	3IVM	5N4F	5N4C	5T88
Conformation	Interm.	Open	Closed	Open	Closed	Open	Interm.	Interm.
Protein	PSP	TbOpB		ApPEP		GmPEP		PfPEP
Residues # (in the crystal structure)*	677	712	710	669	682	703	720	618
Aligned res. #*	677	668	665	605	650	517	659	600
Z-score *	61.8	44.0	46.3	42.5	41.1	39.6	41.6	37.8
Identity, %*	100	37	38	27	27	22	21	22
RMSD, Å*	0	3.8	2.2	4.5	2.8	4.0	2.6	3.0
Catalytic Ser-His Cα-distance, Å	18.2	18.5	8.3	N/a **	8.3	N/a **	15.6	23.6
Cat. S-OG Cat. H-NE2 distance, Å	13.9	18.3	3.5	N/a **	3.3	N/a **	N/a ***	17.4
Catalytic Asp-His Cα-distance, Å	10.6	7.6	4.5	N/a **	4.5	N/a **	8.4	10.9
Cat. D-OD2 Cat. H-ND1, distance, Å	9.0	11.8	3.1	N/a **	2.9	N/a **	10.6	7.0
Center of mass distance, Å	32.3	36.7	30.4	38.7	30.7	39.4	32.0	30.9
Buried surface area, cat./prop. domain, %^1^	11.3/9.4	8.4/7.5	14.0/12.3	8.1/7.7	16.9/14.6	7.5/7.0	13.5/11.4	12.2/11.5
Interfaceresidues, cat./prop. domain, %^2^	16.3/15.9	10.3/10.5	17.4/16.9	12.1/7.7	22.4/19.5	11.0/6.3	19.0/15.2	18.5/15.1
Δ^i^G, kcal/M	−12.9	−8.9	−17.8	−20.8	−23.6	−16.3	−24.3	−23
Hydrogen bonds	11	14	28	7	22	9	25	16
Salt Bridges	4	4	4	-	2	-	1	1

*—according to the Dali structural analysis [41]; **—not available due to the poor electron densities in the His-loop area; ***—not available due to alanine substitution of catalytic Ser; ^1^—percentage of the buried surface area over the total surface area of the domain; ^2^—percentage of residues in the interface over the total residues in the domain.

**Table 4:** SAXS parameters for PSP variants.

Proteins	Rg (Å) (Guinier Approximation)	Dmax (Å) (P(r) Function)	Volume-of-Correlation V(c)
PSP	27.4	80	433
PSP-Sp	27.2	76	434
PSPmodE125A	26.5	80	397
PSPmod	25.9	79	422

**Table 5:** Chi squares (χ^2^) for the comparison of experimental SAXS profile with theoretical generated by FOXS/CRYSOL programs for the models of PSP structures.

Proteins	χ^2^ 7OB1 (FOXS/CRYSOL)	χ^2^ Open	χ^2^ Close
PSP	98.8/65.8	6.1 (6.2)	143.0 (122.9)
PSP-Sp	25.6/7.8	48.1 (51.1)	56.3 (37.6)
PSPmodE125A	25.1/14.3	18.5 (23.2)	51.6 (36.0)
PSPmod	124.1/76.1	25.6 (30.3)	205.0 (163.8)

## Data Availability

The structural data have been deposited to the Protein Data Bank under accession codes 7OB1, 7NE4, 7NE5.

## Supplementary Materials

The following are available online at https://www.mdpi.com/article/10.3390/biology10101021/s1, Figure S1. The real-space correlation coefficient plots for 7OB1, 7NE4 and 7NE5 PDB entries obtained using OVERLAPMAP software from the CCP4 software suite. Figure S2. The tertiary structure of OpB is represented as a scheme, which highlights the domain swap in the catalytic domain, Figure S3: Superposition of PSPmod (PDB 7OB1), PSPmodE125A (PDB ID 7NE4) and PSPmodS532A (PDB ID7NE5), Table S1: Catalytic triad and domains positioning in the crystal structure of PSPmod, PSPmodE125A and PSPmodE75 compared to those in the homology models of wild-type PSP in open and closed conformations and in the model of EcOpB obtained by alphafold.

## References

[1] Polgár L. (2002). The prolyl oligopeptidase family. Cell. Mol. Life Sci..

[2] Rawlings N.D., Barrett A.J., Thomas P., Huang X., Bateman A., Finn R.D. (2018). The MEROPS database of proteolytic enzymes, their substrates and inhibitors in 2017 and a comparison with peptidases in the PANTHER database. Nucleic Acids Res..

[3] Motta F.N., Azevedo C.D.S., Neves B.P., de Araújo C.N., Grellier P., de Santana J.M., Bastos I.M.D. (2019). Oligopeptidase B, a missing enzyme in mammals and a potential drug target for trypanosomatid diseases. Biochimie.

[4] Morty R.E., Pellé R., Vadász I., Uzcanga G.L., Seeger W., Bubis J. (2005). Oligopeptidase B from Trypanosoma evansi. A parasite peptidase that inactivates atrial natriuretic factor in the bloodstream of infected hosts. J. Biol. Chem..

[5] Coetzer T.H., Goldring J.P.D., Huson L.E. (2008). Oligopeptidase B: A processing peptidase involved in pathogenesis. Biochimie.

[6] Swenerton R.K., Zhang S., Sajid M., Medzihradszky K.F., Craik C.S., Kelly B.L., McKerrow J.H. (2011). The Oligopeptidase B of Leishmania Regulates Parasite Enolase and Immune Evasion. J. Biol. Chem..

[7] Bivona A.E., Alberti A.S., Matos M.N., Cerny N., Cardoso A.C., Morales C., González G., Cazorla S.I., Malchiodi E.L. (2018). Trypanosoma cruzi 80 kDa prolyl oligopeptidase (Tc80) as a novel immunogen for Chagas disease vaccine. PLoS Negl. Trop. Dis..

[8] Kanatani A., Masuda T., Shimoda T., Misoka F., Lin X.S., Yoshimoto T., Tsuru D. (1991). Protease II from Escherichia coli: Sequencing and Expression of the Enzyme Gene and Characterization of the Expressed Enzyme1. J. Biochem..

[9] Mattiuzzo M., De Gobba C., Runti G., Mardirossian M., Bandiera A., Gennaro R., Scocchi M. (2014). Proteolytic Activity of Escherichia coli Oligopeptidase B Against Proline-Rich Antimicrobial Peptides. J. Microbiol. Biotechnol..

[10] Fülöp V., Böcskei Z., Polgár L. (1998). Prolyl Oligopeptidase: An Unusual β-Propeller Domain Regulates Proteolysis. Cell.

[11] Rea D., Fülöp V. (2006). Structure-Function Properties of Prolyl Oligopeptidase Family Enzymes. Cell Biophys..

[12] Shan L., Mathews I.I., Khosla C. (2005). Structural and mechanistic analysis of two prolyl endopeptidases: Role of interdomain dynamics in catalysis and specificity. Proc. Natl. Acad. Sci. USA.

[13] Li M., Chen C., Davies D.R., Chiu T.K. (2010). Induced-fit Mechanism for Prolyl Endopeptidase. J. Biol. Chem..

[14] Harmat V., Domokos K., Menyhárd D.K., Palló A., Szeltner Z., Szamosi I., Beke-Somfai T., Náray-Szabó G., Polgár L. (2011). Structure and Catalysis of Acylaminoacyl Peptidase: Closed and open subunits of a dimer oligopeptidase. J. Biol. Chem..

[15] Menyhárd D.K., Orgován Z., Szeltner Z., Szamosi I., Harmat V. (2015). Catalytically distinct states captured in a crystal lattice: The substrate-bound and scavenger states of acylaminoacyl peptidase and their implications for functionality. Acta Crystallogr. Sect. D Biol. Crystallogr..

[16] Szeltner Z., Rea D., Juhász T., Renner V., Fülöp V., Polgár L. (2004). Concerted Structural Changes in the Peptidase and the Propeller Domains of Prolyl Oligopeptidase are Required for Substrate Binding. J. Mol. Biol..

[17] Kichik N., Tarragó T., Claasen B., Gairí M., Millet O., Giralt E. (2011). 15N Relaxation NMR Studies of Prolyl Oligopeptidase, an 80 kDa Enzyme, Reveal a Pre-existing Equilibrium between Different Conformational States. ChemBioChem.

[18] Fülöp V., Szeltner Z., Polgár L. (2000). Catalysis of serine oligopeptidases is controlled by a gating filter mechanism. EMBO Rep..

[19] St-Pierre J.-F., Karttunen M., Mousseau N., Róg T., Bunker A. (2011). Use of Umbrella Sampling to Calculate the Entrance/Exit Pathway for Z-Pro-Prolinal Inhibitor in Prolyl Oligopeptidase. J. Chem. Theory Comput..

[20] Kaushik S., Sowdhamini R. (2011). Structural Analysis of Prolyl Oligopeptidases Using Molecular Docking and Dynamics: Insights into Conformational Changes and Ligand Binding. PLoS ONE.

[21] Szeltner Z., Juhász T., Szamosi I., Rea D., Fülöp V., Módos K., Juliano L., Polgár L. (2013). The loops facing the active site of prolyl oligopeptidase are crucial components in substrate gating and specificity. Biochim. Biophys. Acta (BBA)-Proteins Proteom..

[22] Kaszuba K., Róg T., Danne R., Canning P., Fülöp V., Juhász T., Szeltner Z., Pierre J.-F.S., García-Horsman A., Männistö P.T. (2012). Molecular dynamics, crystallography and mutagenesis studies on the substrate gating mechanism of prolyl oligopeptidase. Biochimie.

[23] Kaushik S., Etchebest C., Sowdhamini R. (2014). Decoding the structural events in substrate-gating mechanism of eukaryotic prolyl oligopeptidase using normal mode analysis and molecular dynamics simulations. Proteins.

[24] Czekster C.M., Ludewig H., McMahon S.A., Naismith J.H. (2017). Characterization of a dual function macrocyclase enables design and use of efficient macrocyclization substrates. Nat. Commun..

[25] Ellis-Guardiola K., Rui H., Beckner R.L., Srivastava P., Sukumar N., Roux B., Lewis J.C. (2019). Crystal Structure and Conformational Dynamics of Pyrococcus furiosus Prolyl Oligopeptidase. Biochemistry.

[26] Canning P., Rea D., Morty R.E., Fülöp V. (2013). Crystal Structures of Trypanosoma brucei Oligopeptidase B Broaden the Paradigm of Catalytic Regulation in Prolyl Oligopeptidase Family Enzymes. PLoS ONE.

[27] McLuskey K., Paterson N.G., Bland N.D., Isaacs N.W., Mottram J. (2010). Crystal Structure of Leishmania major Oligopeptidase B Gives Insight into the Enzymatic Properties of a Trypanosomatid Virulence Factor. J. Biol. Chem..

[28] Mikhailova A.G., Rakitina T.V., Timofeev V.I., Karlinsky D.M., Korzhenevskiy D.A., Agapova Y., Vlaskina A.V., Ovchinnikova M.V., Gorlenko V.A., Rumsh L.D. (2017). Activity modulation of the oligopeptidase B from Serratia proteamaculans by site-directed mutagenesis of amino acid residues surrounding catalytic triad histidine. Biochimie.

[29] Petrenko D.E., Mikhailova A.G., Timofeev V.I., Agapova Y., Karlinsky D.M., Komolov A.S., Korzhenevskiy D.A., Vlaskina A.V., Rumsh L.D., Rakitina T.V. (2020). Molecular dynamics complemented by site-directed mutagenesis reveals significant difference between the interdomain salt bridge networks stabilizing oligopeptidases B from bacteria and protozoa in their active conformations. J. Biomol. Struct. Dyn..

[30] Yan J.-B., Wang G.-Q., Du P., Zhu D.-X., Wang M.-W., Jiang X.-Y. (2006). High-level expression and purification of Escherichia coli oligopeptidase B. Protein Expr. Purif..

[31] Morty R.E., Fülöp V., Andrews N.W. (2002). Substrate Recognition Properties of Oligopeptidase B from Salmonella enterica Serovar Typhimurium. J. Bacteriol..

[32] Mikhailova A.G., Khairullin R.F., Demidyuk I.V., Kostrov S.V., Grinberg N.V., Burova T.V., Grinberg V.Y., Rumsh L.D. (2014). Cloning, sequencing, expression, and characterization of thermostability of oligopeptidase B from Serratia proteamaculans, a novel psychrophilic protease. Protein Expr. Purif..

[33] Boyko K.M., Rakitina T.V., Korzhenevskiy D.A., Vlaskina A.V., Agapova Y.K., Kamashev D.E., Kleymenov S.Y., Popov V. (2016). Structural basis of the high thermal stability of the histone-like HU protein from the mollicute Spiroplasma melliferum KC3. Sci. Rep..

[34] Petrenko D.E., Nikolaeva A.Y., Lazarenko V.A., Dorovatovskii P.V., Timofeev V., Vlaskina A.V., Korzhenevskiy D.A., Mikhailova A.G., Rakitina T.V. (2020). Screening of Conditions that Facilitate Crystallization of Oligopeptidase B from Serratia Proteamaculans by Differential Scanning Fluorimetry. Crystallogr. Rep..

[35] Petrenko D.E., Nikolaeva A.Y., Lazarenko V.A., Dorovatovskiy P.V., Timofeev V.I., Vlaskina A.V., Korzhenevskiy D.A., Mikhailova A.G., Boyko K.M., Rakitina T.V. (2020). Crystallographic Study of Mutants and Complexes of Oligopeptidase B from Serratia proteamaculans. Crystallogr. Rep..

[36] Long F., Vagin A.A., Young P., Murshudov G.N. (2008). BALBES: A molecular-replacement pipeline. Acta Crystallogr. Sect. D Biol. Crystallogr..

[37] Murshudov G.N., Skubák P., Lebedev A.A., Pannu N.S., Steiner R.A., Nicholls R., Winn M.D., Long F., Vagin A.A. (2011). REFMAC5 for the refinement of macromolecular crystal structures. Acta Crystallogr..

[38] Emsley P., Lohkamp B., Scott W., Cowtan K.D. (2010). Features and development of Coot. Acta Crystallogr..

[39] Krissinel E., Henrick K. (2007). Inference of Macromolecular Assemblies from Crystalline State. J. Mol. Biol..

[40] Collaborative Computational Project Number 4 (1994). The CCP4 suite: Programs for protein crystallography. Acta Cryst..

[41] Holm L. (2020). Using Dali for Protein Structure Comparison. Methods Mol. Biol..

[42] Manalastas-Cantos K., Konarev P.V., Hajizadeh N.R., Kikhney A.G., Petoukhov M.V., Molodenskiy D.S., Panjkovich A., Mertens H.D.T., Gruzinov A., Borges C. (2021). ATSAS 3.0: Expanded functionality and new tools for small-angle scattering data analysis. J. Appl. Crystallogr..

[43] Hopkins J.B., Gillilan R.E., Skou S. (2017). BioXTAS RAW: Improvements to a free open-source program for small-angle X-ray scattering data reduction and analysis. J. Appl. Crystallogr..

[44] Svergun D. (1992). Determination of the regularization parameter in indirect-transform methods using perceptual criteria. J. Appl. Crystallogr..

[45] Rambo R.P., Tainer J. (2013). Accurate assessment of mass, models and resolution by small-angle scattering. Nature.

[46] Svergun D.I. (1999). Restoring Low Resolution Structure of Biological Macromolecules from Solution Scattering Using Simulated Annealing. Biophys. J..

[47] Pettersen E.F., Goddard T.D., Huang C.C., Couch G.S., Greenblatt D.M., Meng E.C., Ferrin T. (2004). UCSF Chimera? A visualization system for exploratory research and analysis. J. Comput. Chem..

[48] Schneidman-Duhovny D., Hammel M., Tainer J., Sali A. (2013). Accurate SAXS Profile Computation and its Assessment by Contrast Variation Experiments. Biophys. J..

[49] Svergun D., Barberato C., Koch M.H.J. (1995). CRYSOL– a Program to Evaluate X-ray Solution Scattering of Biological Macromolecules from Atomic Coordinates. J. Appl. Crystallogr..

[50] Mikhailova A.G., Nekrasov A.N., Zinchenko A.A., Rakitina T.V., Korzhenevsky D.A., Lipkin A.V., Razguljaeva O.A., Ovchinnikova M.V., Gorlenko V.A., Rumsh L.D. (2015). Truncated variants of Serratia proteamaculans oligopeptidase B having different activities. Biochemestry (Moscow).

[51] Ovchinnikova M.V., Mikhailova A.G., Karlinsky D.M., Gorlenko V.A., Rumsh L.D. (2018). Reversible Cyclic Thermal Inactivation of Oligopeptidase B from Serratia proteamaculans. Acta Nat..

[52] Farhadian S., Shareghi B., Saboury A.A. (2016). Exploring the thermal stability and activity of α-chymotrypsin in the presence of spermine. J. Biomol. Struct. Dyn..

[53] Jumper J., Evans R., Pritzel A., Green T., Figurnov M., Ronneberger O., Tunyasuvunakool K., Bates R., Žídek A., Potapenko A. (2021). Highly accurate protein structure prediction with AlphaFold. Nature.

[54] Fukumoto J., Ismail N.I.M., Kubo M., Kinoshita K., Inoue M., Yuasa K., Nishimoto M., Matsuki H., Tsuji A. (2013). Possible role of inter-domain salt bridges in oligopeptidase B from Trypanosoma brucei: Critical role of Glu172 of non-catalytic -propeller domain in catalytic activity and Glu490 of catalytic domain in stability of OPB. J. Biochem..

