# Supplementary material for: First Crystal Structure of Bacterial Oligopeptidase B in an Intermediate State: The Roles of the Hinge Region Modification and Spermine
Source: Biology (Basel). 2021 Oct 9;10(10):1021. doi: 10.3390/biology10101021 (PMC8533160; doi:10.3390/biology10101021)
Supplement: Supplementary file 1 [file biology-10-01021-s001.zip › biology-1377326-supplementary.pdf]

Supplementary Information

First crystal structure of bacterial oligopeptidase B in an intermediate state: the roles of the hinge region modification and spermine.

Dmitry E. Petrenko, Vladimir I. Timofeev, Vladimir V. Britikov, Elena V. Britikova, Sergey Y. Kleymenov, Anna V. Vlaskina, Inna P. Kuranova, Anna G. Mikhailova and Tatiana V. Rakitina

Figure S1 2

Figure S2 3

Table S1 4

Figure S3 5

References 6

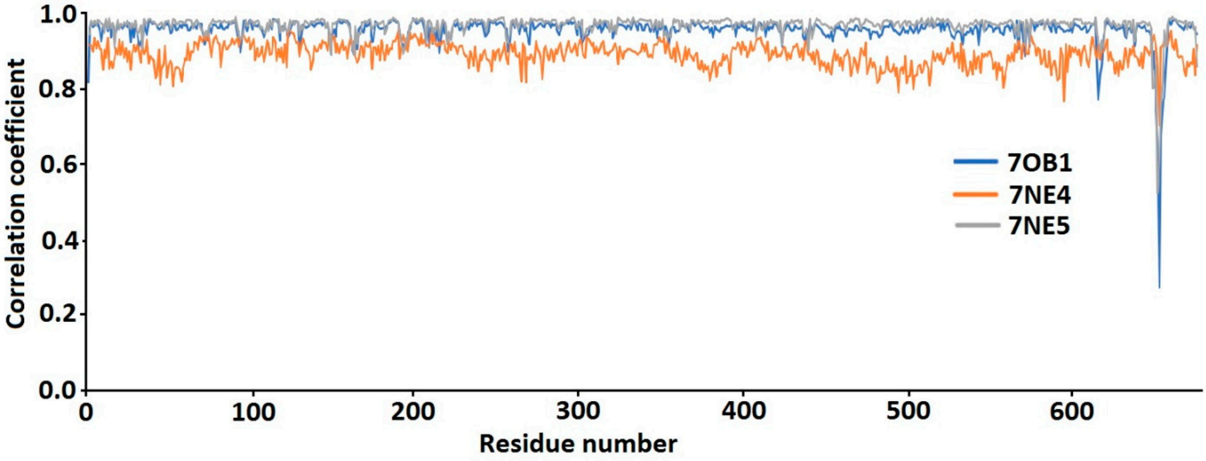

Figure S1. The real-space correlation coefficient plots for 7OB1, 7NE4 and 7NE5 PDB entries obtained using OVERLAPMAP software [1, 2] from the CCP4 software suite [3].

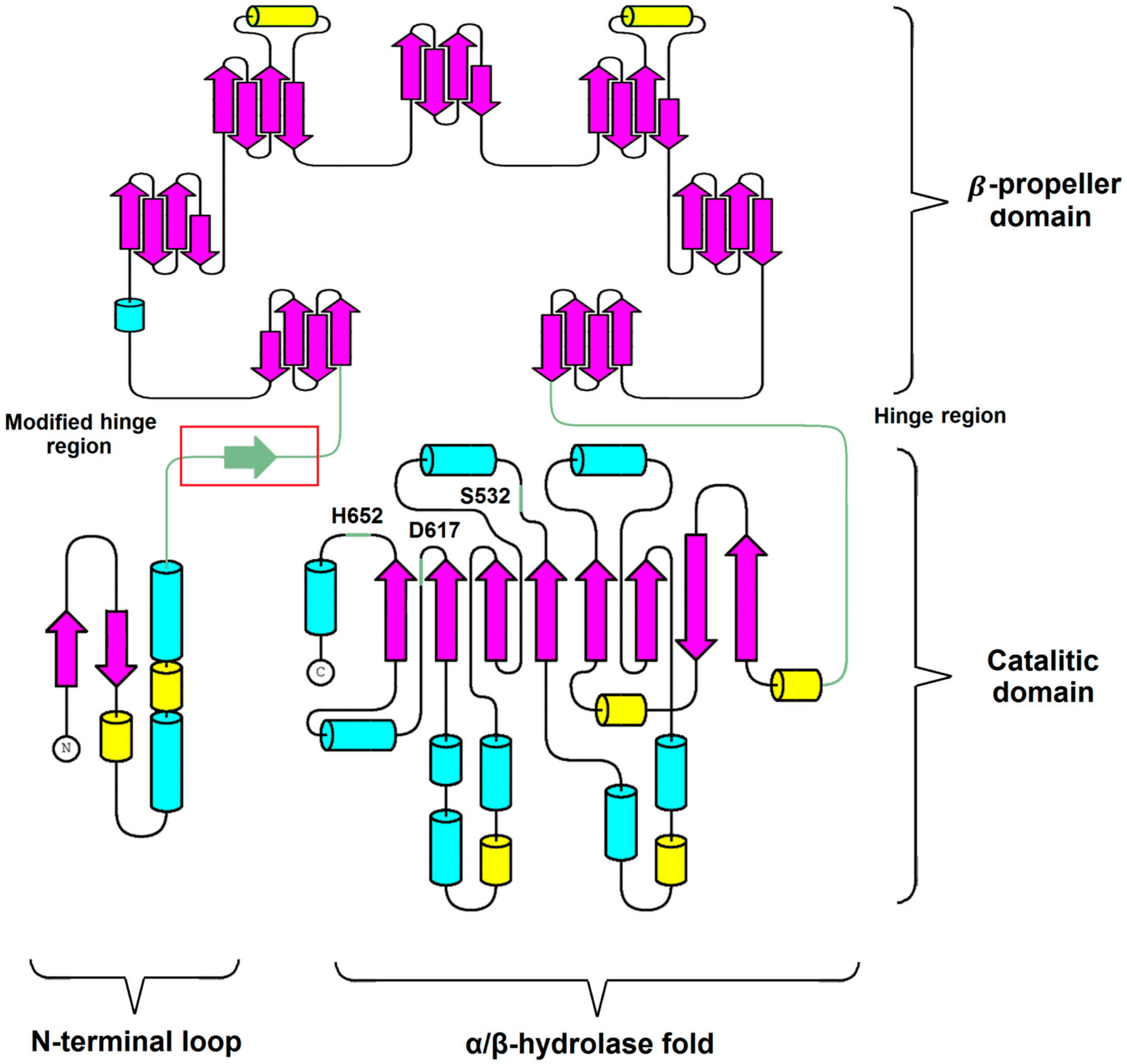

Figure S2. The tertiary structure of OpB is represented as a scheme, which highlighted the domain swap in the catalytic domain. The topology was prepared with TOPDRAW [4] using crystal structure of PSPmod. In the catalytic and β-propeller domains, loop regions are shown in black, α-helices in cyan, β-strands in magenta, and 3_{10} helices in yellow. The hinge regions between the two domains and the catalytic triad are shown in pale green. The numeration of the catalytic triad residues is according to the PSP sequence. The residues of the first hinge peptide modified in PSPmod are in red square.

Table S1. Catalytic triad and domains positioning in the crystal structures of PSPmod, PSPmodE125A and PSPmodE75 compared to those in the homology models of wild type PSP in open and closed states and the model of OpB from *E. Coli* (EcOpB) predicted by Alphafold [6].

	Crystal structures			Homology models		
PDB ID/model	7OB1	7NE4	7NE5	Open[3]	Closed[3]	Alphafold[4]
Protein	PSPmod	PSPmod E125A	PSPmod S532A	PSP	PSP	EcOpB
RMSD (Å)	0	0.9	0.6	4.1	2.0	0.8
S532-H652 Cα-distance (Å)	18.2	18.4	19.4*	18.8	8.6	8.1
S532OG-H652NE2 Distance (Å)	13.9	22.5	N/a	16.7	2.8	3.1
Asp617-His652 Cα-distance (Å)	10.6	9.7	11.2	8.0	4.8	4.6
Asp617-OD2-His652-ND1 Distance (Å)	9.0	12.6	13.9	11.7	2.9	2.7
Center mass distance, (Å)	32.3	31.6	32.0	37.1	30.3	31.4
Buried surface area, cat./prop. Domain (%)[1]	11.3/9.4	13.0/10.7	11.8/10.0	6.9/6.8	14.8/12.5	15.2/13.1
Interface residues, cat./prop. domain (%)[2]	16.3/15.9	17.6/16.2	17.0/14.7	10.7/9.7	20.5/18.2	20.2/18.0
$\Delta^{i}G$ (kcal/M)	-12.9	-16.7	-16.9	-12.1	-21.1	-19.1
Hydrogen bonds	11	17	14	10	19	21
Salt Bridges	4	4	4	2	3	3

*For A532-H652

[1] - percentage of the buried surface area over the total surface area of the domain.

[2] - percentage of residues in the interface over the total residues in the domain

[3] - the data was taken from [5]

[4] - the data was taken from https://alphafold.ebi.ac.uk/entry/P24555

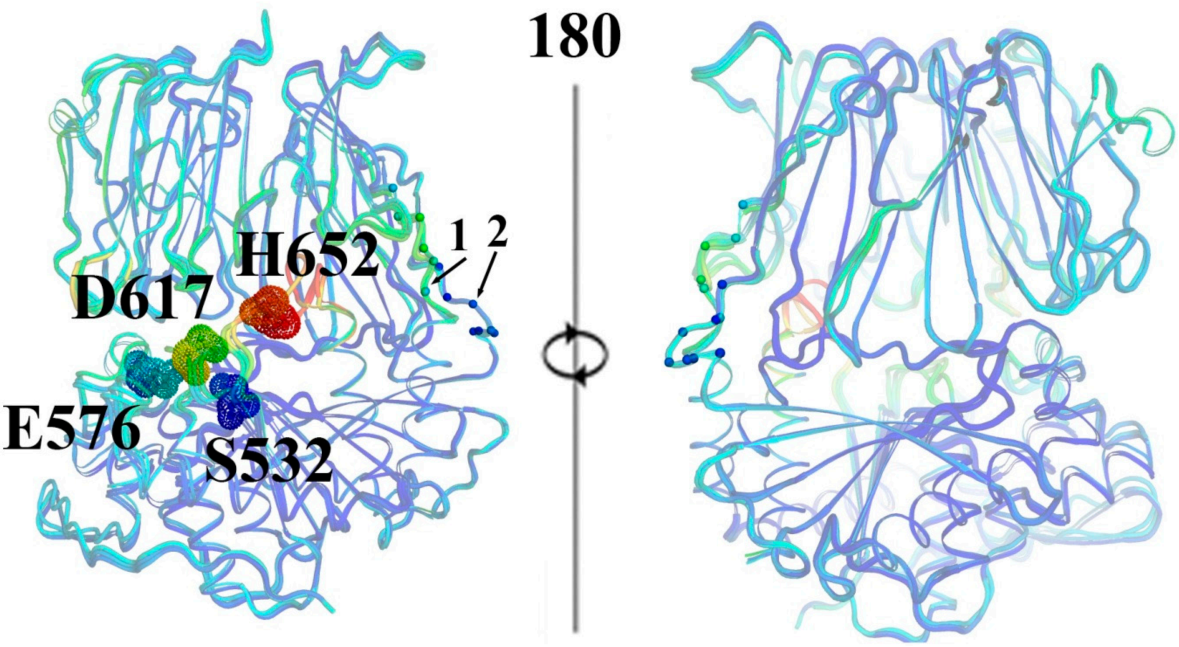

Figure S3. Superposition PSPmod (PDB ID 7OB1), PSPmodE125A (PDB ID 7NE4) PSPmodS532A (PDB ID 7NE5). Schematic representations of the structures are color-ramped blue to red from low to high residual B-factors. The catalytic triad and S1 substrate-binding center residues of PSPmod are in dotted representations. The hinge peptides are numbered and their residues are shown as balls. The three-dimensional figures were produced using PyMOL Molecular Graphics System, Version 1.9.0.0 (Schrödinger, USA).

Supplementary references

1. Brändén C., Jones T. A. Between objectivity and subjectivity. Nature (London), 1990. 343, 687–689; DOI: 10.1038/343687a0

2. Jones Y., Stuart D. Proceedings of the CCP4 Study Weekend. Isomorphous Replacement and Anomalous Scattering, edited by W. Wolf, P. R. Evans & A. G. W. Leslie. Warrington: Daresbury Laboratory. 1991. 39–48.

3. Winn M.D., Ballard C.C., Cowtan K.D., Dodson E.J., Emsley P., Evans P.R., Keegan R.M., Krissinel E.B., Leslie A.G., McCoy A., McNicholas S.J., Murshudov G.N., Pannu N.S., Potterton E.A., Powell H.R., Read R.J., Vagin A., Wilson K.S.. Overview of the CCP4 suite and current developments. Acta Crystallogr D Biol Crystallogr. 2011, 67(4), 235-42. doi: 10.1107/S0907444910045749

4. Bond C.S. TopDraw: A sketchpad for protein structure topology cartoons. Bioinformatics. 2003; 19, 311-312 https://doi.org/10.1093/bioinformatics/19.2.311

5. Mikhailova A.G., Rakitina T.V., Timofeev V.I., Karlinsky D.M., Korzhenevsky D.A., Agapova Yu. K., Vlaskina A.V., Ovchinnikova M.V., Gorlenko V.A., Rumsh L.D. Activity modulation of the oligopeptidase B from Serratia pro-teamaculans by site-directed mutagenesis of amino acid residues surrounding catalytic triad histidine. Biochimie. 2017; 139:125–136. http://dx.doi.org/10.1016/j.biochi.2017.05.013

6. Jumper J., Evans R., Pritzel A., Green T., Figurnov M., Ronneberger O., Tunyasuvunakool K., Bates R., Žídek A, Potapenko A et al. Highly accurate protein structure prediction with AlphaFold. Nature. 2021; 596: 583–589. https://doi.org/10.1038/s41586-021-03819-2
